# Animal–Visitor Interactions in Zoos and Aquariums: A Systematic Review

**DOI:** 10.3390/ani15131924

**Published:** 2025-06-29

**Authors:** Ga-Yi Lin, Keith Chi Hui Ng, Eduardo J. Fernandez

**Affiliations:** School of Animal and Veterinary Sciences, The University of Adelaide, Adelaide, SA 5371, Australia; ga-yi.lin@student.adelaide.edu.au (G.-Y.L.); keith.ng@student.adelaide.edu.au (K.C.H.N.)

**Keywords:** animal-visitor interactions, human-animal interactions, zoos, aquariums, visitor effects, visitor experiences

## Abstract

There is increasing recognition of the importance of human–animal interaction (HAI) research in the assessment of animal welfare. This is partly reflected by the appearance of increasing animal–visitor interaction (AVI) publications in zoos. Early AVI publications primarily focused on primates and the impact of visitors on animals (visitor effects), with most identifying negative welfare impacts. This review aims to identify trends and changes in the key factors of AVIs (e.g., types of interactions measured; taxa studied; welfare impact). Covidence online software and PRISMA were used to screen papers and extract data. A total of 157 papers comprising 314 studies were included. AVI publications have increased in the last two decades, with a large increase in publications since 2020. Most publications have focused on visitor effects, as opposed to the impact of animals on visitors (visitor experiences). Earlier visitor effect studies mostly focused on primates, while more recent studies have primarily focused on non-primate species. Excluding studies categorized as neutral, there were more visitor effect studies with measures focused on negative than positive welfare impacts. However, the last decade and a half has seen a substantial increase in visitor effect studies measuring positive welfare impacts. These results are discussed in reference to growing changes in the types of studies, species, and outcomes measured in AVIs. In addition, we consider the future of AVI research, including a growing need for and consideration of standardized welfare assessments, the increased use of experimental control, and AVIs that equally promote visitor education and positive animal welfare.

## Simple Summary

Animal–visitor interactions (AVIs) have become increasingly important for zoo research. However, little progress has been made to systematically evaluate publications related to the impact of both visitors on animals (visitor effects) and animals on visitors (visitor experiences). This systematic review examined impacts over time, particularly in relation to differences between visitor effects and visitor experiences, primate and non-primate visitor effect studies, and positive and negative welfare impacts measured in visitor effect studies. Overall, more recently, research has continued to emphasize visitor effect publications over visitor experience publications. However, recent publications have also focused more on non-primate species and positive welfare effects in visitor effect studies. The results are discussed with respect to the growing evidence for the benefits of animal–visitor interactions in zoos, as well as the future needs of AVI research.

## 1. Introduction

Human–animal interactions (HAIs) are a core component for understanding the welfare of animals [1,2,3]. Zoos and aquariums have spent considerable time studying HAIs in the form of animal–visitor interactions (AVIs) [4]. Early AVI research was primarily focused on visitor effects or the impact of the visitor on the welfare of the animal [5,6]. Many visitor effect studies have highlighted the negative welfare impacts on captive animals, particularly primates in zoos [6,7,8]. However, more recent publications have expanded to include visitor effects with positive welfare impacts [9,10] and non-primate species [11], as well as focusing on the impact of AVIs on the visitor, or ‘the visitor experience’ [12,13,14].

AVI research has typically been divided into two main areas of focus, as noted above: (1) the impact visitors have on the captive animals, or ‘the visitor effect’, and (2) the impact captive animals have on the visitors, or ‘the visitor experience’ [15]. The impact of visitors on zoo animals has been studied extensively by assessing behavioral and physiological changes in animals in response to varying visitor conditions [4,5,9]. The welfare impact on exhibited animals is typically categorized as negative, positive, or neutral [6]. A negative impact is when the visitor acts as a source of stress to the animals, which is manifested in observable signs such as increased stereotypical behaviors, heightened visitor avoidance or vigilance, or increased physiological markers for stress. Visitors serving as a positive impact on zoo animals are typically indicated by animals displaying heightened interest in visitors or visitor areas and actively moving closer to visitors or seeking visitors’ attention. The absence of behavioral or physiological changes is a case where visitors have no noticeable or neutral influence on the exhibited animal [5]. In the context of AVIs, visitor experiences explore how visitor perceptions, attitudes, and behaviors toward zoo animals are influenced by exhibited animals and their behavior [13].

Overall, the underlying mechanisms and purpose of AVI research have focused on two major components: The welfare impact of visitor effects on the zoo-housed animals and the educational impact of interactions on the visitor experience. The former visitor effects are typically studied in terms of visitor presence or absence [16], with additional factors such as visitor intensity (e.g., type of visitor activity; overall volume) occasionally studied [17,18]. The latter visitor experience has been studied in terms of both impact on visitor activity (e.g., crowd size; length of visitor stay) as well as perceptions and responses to surveys [13]. In addition, the impact of interactions on visitors has been studied not just in terms of educational and entertainment aspects but also how the visitor experience might impact conservation efforts, including financial support outside of the zoo [12,19,20]. Therefore, the goal of many research efforts is to better understand and optimize interactions for both animals and visitors.

To date, ten major reviews on the topic of AVIs in zoos and aquariums have been conducted [4,5,6,7,9,11,12,13,14,21], with the majority looking at visitor effects. Of these reviews, Williams et al. [11] was the first to conduct a non-narrative review; however, it was limited to visitor effects on non-primate animals in zoos. To our knowledge, a systematic review of AVIs that includes both primates and non-primates and examines both visitor effects and experiences has not previously been performed. Our review focuses on three key aspects of AVIs over time: (a) the type of interactions measured (visitor effects; visitor experiences), (b) the taxa studied (primate; non-primate), and (c) welfare impact (positive; negative). The aim is to identify emerging trends within the AVI research field regarding the aforementioned key factors of AVI studies over time. In addition, we tested three predictions (hypotheses) with respect to the factors noted above: (1) visitor experience studies have increased over time proportionally compared to visitor effect studies, (2) non-primate visitor effect studies have increased over time proportionally compared to primate visitor effect studies, and (3) the positive measures of animal welfare in visitor effect studies have increased over time proportionally compared to the negative measures of animal welfare in visitor effect studies.

## 2. Materials and Methods

### 2.1. Search Terms and Databases

An extensive search was undertaken to capture a range of relevant papers across multiple databases. To be specific in the search, the main search terms were synthesized using ‘zoos/aquariums’ and ‘visitor interaction’. The following combinations utilizing Boolean and close proximity operators were searched on Scopus, Web of Science, Zoological Record, and Proquest, based on the following categories:

**(Setting):** Zoos OR aquarium* OR zoo OR safari OR “wildlife conservation” OR sanctuary OR “zoological institution*” OR primate OR non-primate OR “wildlife park*” OR “zoological park*” OR “bird park*” OR aviary
AND
**(Topic):** “Human-animal interaction*” OR “animal-visitor interaction*” OR “human-animal relationship*” OR “animal-visitor relationship*” OR visitor* OR ((Visitor* OR visiting OR visit*) near/4 (Impact* OR effect* OR behaviour* OR behavior* OR relationship* OR interaction*)) OR HAI OR AVI OR “visitor effect*” OR “visitor experience*”.

PubMed was also searched using the following combination:

**(Setting):** “Animals, Zoo”[mh] OR Zoos[tiab] OR aquarium*[tiab] OR zoo[tiab] OR safari[tiab] OR wildlife conservation[tiab] OR sanctuary[tiab] OR zoological institution*[tiab] OR primate[tiab] OR non-primate[tiab] OR wildlife park*[tiab] OR zoological park*[tiab] OR bird park* [tiab] OR aviary [tiab]
AND
**(Topic):** “Human–Animal Interaction”[mh:noexp] OR Human–Animal interaction*[tiab] OR animal–visitor interaction*[tiab] OR Human–Animal relationship*[tiab] OR animal–visitor relationship*[tiab] OR visitor*[tiab] OR “visitor experience*”.

In addition, zoo-specific journals sometimes missed in the above search engines, such as the Journal of Zoological and Botanical Gardens (JZBG) and the Journal of Zoo and Aquarium Research (JZAR), were searched directly via their websites. The search terms used for JZBG were the same as the first combinations for the multiple databases, with the exception of the proximity operators to accommodate the databases’ advanced search settings. As JZAR did not have an advanced search function, the following terms were searched separately: ‘visitor impact’, ‘visitor effect’, ‘visitor experience’, ‘animal visitor interaction’, ‘human animal interaction’, and ‘visitor’. The search terms used were refined by terminology commonly used in this field, while considering country-specific animal facilities to capture the relevant literature globally. Searches were also restricted to titles, abstracts, and keywords, while being filtered by language (English) and peer-reviewed articles. All records were imported into the Covidence online software (https://www.covidence.org/, accessed on 15 August 2023), which is one of the standard systematic review tools available. Covidence allowed us to import and organize papers, including duplicates which were automatically removed by the system.

### 2.2. Inclusion/Exclusion Criteria

**Figure 1:**
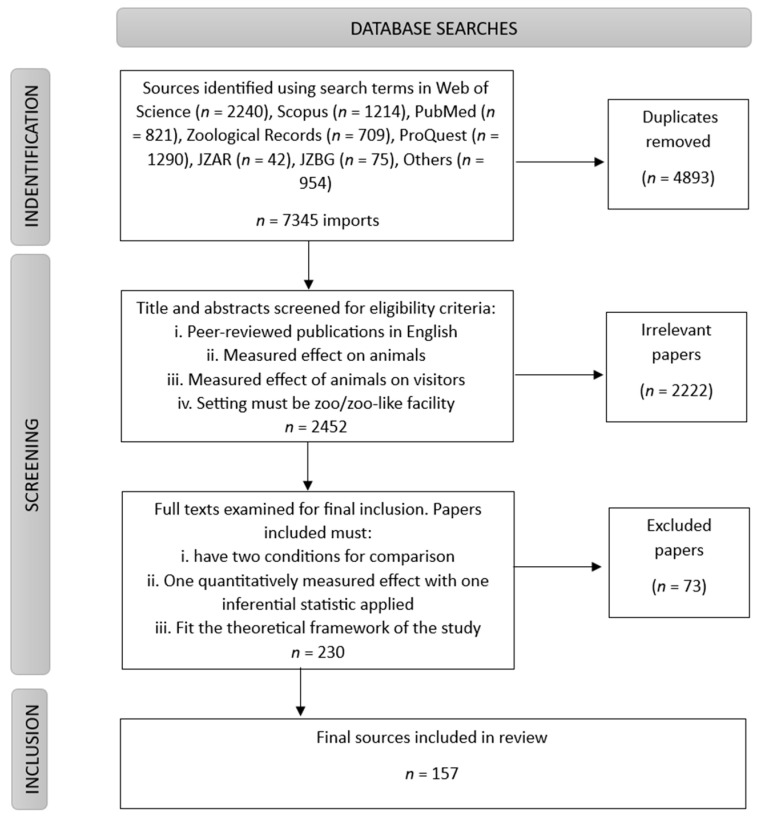
Flowchart outlining the identification and inclusion of the relevant literature.

The aim of this systematic review was to measure three key factors over time: (1) the type of animal–visitor interactions measured (i.e., visitor effects or experiences), (2) taxa, and (3) welfare impact using the Preferred Reporting Items for Systematic Reviews and Meta-Analysis Protocols (PRISMA; [22]). To be included in this review, the papers needed to measure the impact of visitors on animals (visitor effects) or the impact of animals on visitors (visitor experiences). Peer-reviewed research publications were included if they were written in English (e.g., yes = journal; no = preprint; book chapter; non-published material) and had at least two conditions for comparison (e.g., low versus high crowd numbers; low versus high animal aggression). Publications also had to have at least one quantitatively measured effect, with at least one inferential statistic applied (e.g., yes = experiments; observational studies; no = reviews; exclusively qualitative studies). Papers were excluded if they did not fit into the theoretical framework of the study (e.g., results based on wildlife as opposed to captive wild animals in enclosures). Papers that measured data relating to zoos but did not specify an animal, measured keeper–animal interactions, or did not directly measure visitor activity (e.g., simply defined weekends as high visitor numbers and weekdays as low visitor numbers) were also excluded from the final screening. A total of 2388 potential papers were identified in the initial screening. Sixty-four potential papers were identified in the updated search. After applying the inclusion and exclusion criteria, 157 relevant articles were suitable for data extraction in the final review (see Figure 1).

### 2.3. Data Extraction

Sources with publishing dates up to the end of 2023 were imported into Covidence. Data were extracted from the included articles for the following information when applicable: author/s, year of publication, article title, journal, class, order, species, and variables used for measures. In cases where more than one species was recorded within the same paper, they were classified as separate studies and coded individually. The original search was completed on 15 September 2023 and updated on 3 January 2024.

### 2.4. Classification of Metrics

All publications were categorized according to two metrics: papers and studies. Any publication was equated to a single paper. Studies were defined as more than one experiment, observation period, or species examined and could occur as multiple studies per paper. For instance, Hosey and Druck [18] examined visitor effects across 12 species of primates. Therefore, this publication would be categorized as one paper and 12 studies.

Specific metrics were chosen to classify and identify different trends in the studies to allow for comparisons using basic descriptive and inferential statistics. Animal class was categorized as mammal, reptile, bird, amphibian, fish, and other. In addition, on the occasion where data were collectively presented for a group of species, these were classified under the category of other as ‘multi-species’.

#### Binomial Categorization

For all major categories directly compared (i.e., visitor effects and visitor experiences; primate and non-primate species; positive and negative welfare impacts), the metrics were binomially categorized so that the observation of the measure was noted (no = 0; yes = 1). For instance, if a paper measured a visitor effect with at least one positive impact on animal welfare and no neutral or negative welfare impacts, the visitor effect category in the spreadsheet column would list ‘1’ and the visitor experience category would list ‘0’. Similarly, the positive welfare impact category would list ‘1’, and the neutral and negative welfare impact categories would list ‘0’.

It is additionally worth noting that positive and negative welfare impacts were determined in terms of the change observed and its relational value to the overall animal welfare. For example, an increase in foraging or locomotion would likely be viewed as a positive welfare impact, whereas an increase in aggression or stereotypies would likely be viewed as a negative welfare impact. Each study could also include multiple welfare effects, with the possibility of being recorded as simultaneously having a positive, neutral, and negative welfare impact in the same study (up to one for each effect in any one study). Finally, for a welfare impact to be categorized as negative or positive, a statistically significant value must be recorded in the paper, while following the authors’ interpretation of that change (e.g., if increased standing or vigilance was interpreted as behaviorally neutral, this was recorded as such despite recording a significant change). Measured variables with no statistical change were categorized as neutral. Neutral measures were also not included in any analyses, since almost all visitor effect studies (89.2%) included at least one neutral measure.

### 2.5. Hypothesis Testing

The primary focus of this review was to examine descriptive changes over time within published AVI research. Nonetheless, we also proposed three predictions (hypotheses) that were tested with regard to their study occurrence. The first hypothesis involved the types of interactions (visitor effects or visitor experiences) measured, as it related to reviews that have suggested more recent increased interest in visitor experience studies [12,13]:

**H1.**  *Visitor experience studies have increased over time proportionally compared to visitor effect studies.*

Because there was limited published visitor experience research to examine, the latter two hypotheses were specific to visitor effect studies. Specifically, these two hypotheses were related to older reviews stating that visitor effect studies primarily focused on the negative effects of visitors on primates [4,5,6], and newer reviews suggesting that more recent visitor effect studies have focused on both non-primate species and positive welfare effects [9,11]:

**H2.**  *Non-primate visitor effect studies have increased over time proportionally compared to primate visitor effect studies.*

**H3.**  *Positive animal welfare impacts measured in visitor effect studies have increased over time proportionally compared to negative animal welfare impacts measured in visitor effect studies.*

In all three cases, each year was assessed for the percentage of studies identified (see the following results and figures). For instance, 2005 had six studies, with three studies conducted with primate species and three with non-primate species. Therefore, each would be categorized as a 50% occurrence for that year. Hypotheses were then tested by comparing the first half of all years observed (i.e., earlier period) to the latter half of all years observed (i.e., later period). For example, there were 26 years during which a primate or non-primate visitor effect paper was published. Our second primate/non-primate-focused hypothesis was tested by comparing the first half of those years to the latter half (*n* = 13 for both). It is also worth noting that this resulted in different period of time comparisons across the different hypotheses. For instance, although there were 26 years in which a primate or non-primate visitor effect paper was published, there were only 22 years in which a positive or negative visitor effect on welfare was published. Therefore, the comparison years for both would be different (e.g., 1987–2010 and 2011–2023 for earlier- and later-period primate and non-primate comparisons; 1987–2012 and 2013–2023 for earlier- and later-period positive and negative welfare comparisons).

### 2.6. Statistical Analysis

Chi-square goodness of fit tests were used for comparisons between the variables of interest. Specifically, chi-square tests were undertaken to measure the interactions (visitor effects and visitor experiences) at the paper and study level, the frequency of non-primate to primate species in visitor effect studies, the frequency of negative and positive welfare impacts in visitor effect studies, and the frequency of visitor effect studies across animal classes/taxa. All tests were conducted with significance levels set to 0.05, with the exception of multiple comparisons (e.g., tests across multiple taxonomic classes), where Bonferroni corrections were applied. In addition, our three hypotheses were tested by comparing earlier periods to the later periods of time. Normality (Shapiro–Wilk) tests for all three failed, and therefore Mann–Whitney *U* tests were used to analyze the prediction results. SigmaPlot^TM^, version 14.0 (Systat Software Inc., San Jose, CA, USA) was used to create all graphs and run all statistical analyses.

## 3. Results

### 3.1. Papers and Studies over Time

AVI papers in this review were published across 44 journals, with almost half being published in *Zoo Biology* (papers: *n* = 32; studies: *n* = 73), *Applied Animal Behaviour Science* (papers: *n* = 28; studies: *n* = 58), or *Animals* (papers: *n* = 19; studies: *n* = 53).

**Figure 2:**
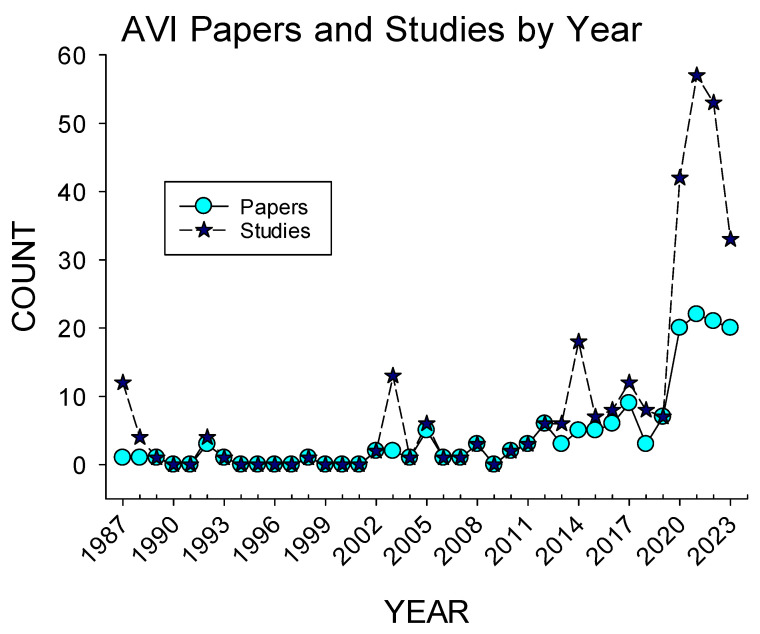
Overview of the number of papers and studies published per year from 1987 to 2023.

Figure 2 shows the number of AVI papers and studies per year. The first publication relating to AVIs was in 1987. Limited papers were published each year (*n* < 3) after this, with the first notable increase occurring in 2005, with five published papers in a single year. A total of six papers were published between 2006 and 2008, before a drop to zero in 2009. Starting in 2010, papers were consistently published every year, until a marked increase in 2017 and 2019, with 10 and seven published papers, respectively. Starting in 2020, there was a large and continued increase in publications, with the total number of publications from 2020 to 2023 summing 84 papers, or slightly more than half of all the papers published to date (53.5%). Figure 2 also demonstrates the number of published studies each year. Overall, 157 papers and 314 studies were included in this review. Out of these 157 papers, 44 (28%) of them recorded more than one study, with the greatest number of studies being sixteen separate species in 2 separate papers [23,24].

### 3.2. AVI Publications During COVID-19

Table 1 shows the relationship between COVID-19 and AVI publications between 2020 and 2023. Previously, researchers had attributed an observed and recent increase in AVI research being published directly to COVID-19-related zoo closures [11]. Our results suggested a similar large increase in AVI publications starting in 2020 (see Figure 1). However, when comparing papers related and unrelated to COVID-19 during the 2020’2023 period (i.e., research completed during the pandemic), non-COVID-19-related papers (*n* = 62) were published 3.1 times more than COVID-19-related papers (*n* = 20), comprising three-quarters (75.6%) of all papers between this period. Papers unrelated to the pandemic dominated in 2020 (*n* = 20) and were higher in all years, with the exception of 2022, where related and non-related papers were equal in number (*n* = 10). In addition, if we exclude the 2020 publications, which only included research conducted prior to the pandemic (2010–2019; see latter Section 4.1), this still resulted in non-COVID-19-related papers (*n* = 42) occurring more than twice as much as the COVID-19-related papers in the same time period (*n* = 20), with non-COVID 19-related papers comprising 67.7% of all papers published between the 2021 and 2023 period.

### 3.3. Visitor Effect and Experience Papers and Studies

Table 2 highlights the differences in interactions covered in both papers and studies that occurred over the years. When separated into visitor effect and visitor experience interactions (Table 2), visitor effects were observed significantly more than visitor experiences in both papers (χ^2^ = 83.062, *df* = 1, *p* < 0.001) and studies (χ^2^ = 183.439, *df* = 1, *p* < 0.001). Five papers that looked at both visitor effect and visitor experience interactions [25,26,27,28,29] were classified separately into both categories. Looking at papers, visitor effects comprised most of these interactions (*n* = 139, 88.5%), while visitor experiences were measured in less than one-fifth of all papers published (*n* = 23, 14.6%), making visitor effects six times more likely to be reported in comparison to visitor experiences.

**Figure 3:**
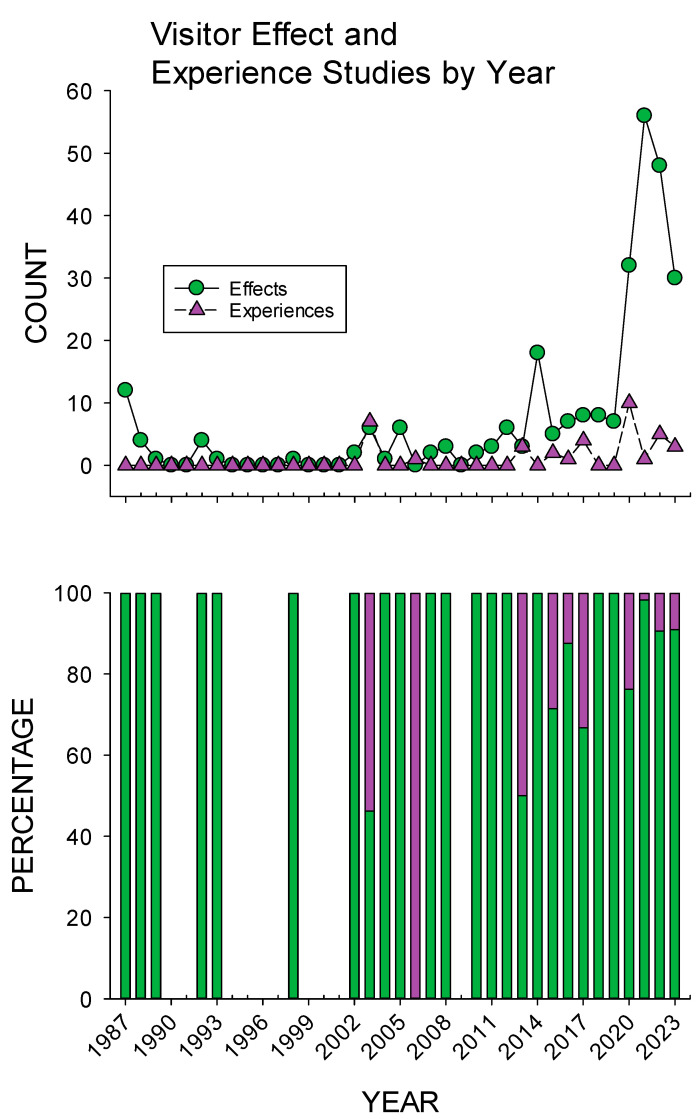
Overview of the changes in visitor effect and experience studies published per year from 1987 to 2023. Note: green = visitor effect studies; purple = visitor experience studies.

Figure 3 shows the count and percentage of visitor effect and experience studies by year. With the exception of several years, visitor effect studies were the majority of AVIs measured. However, beginning in 2013, visitor experiences have become a regularly measured AVI. In testing our first hypothesis that visitor experience studies increased over time proportionally compared to visitor effect studies, we compared the earlier period (1987–2010, *n* = 14) to the later period (2011–2023, *n* = 13; see Section 2.5. for earlier- and later-period descriptions), with no significant difference demonstrated (*U* = 56.0, *p* = 0.053). Overall, visitor experience studies increased proportionally from a mean of 11.0% (SE = 7.8) in the earlier period to a mean of 13.0% (SE = 4.5) in the latter period.

### 3.4. Primate and Non-Primate Visitor Effect Studies

When visitor effect studies were separated into primate and non-primate categories, there were significantly more non-primate studies (χ^2^ = 29.681, *df* = 1, *p* < 0.001). Approximately one-third of all studies focused on primates (33.9%, *n* = 94), with the remainder focusing on non-primate species (66.1%, *n* = 183).

**Figure 4:**
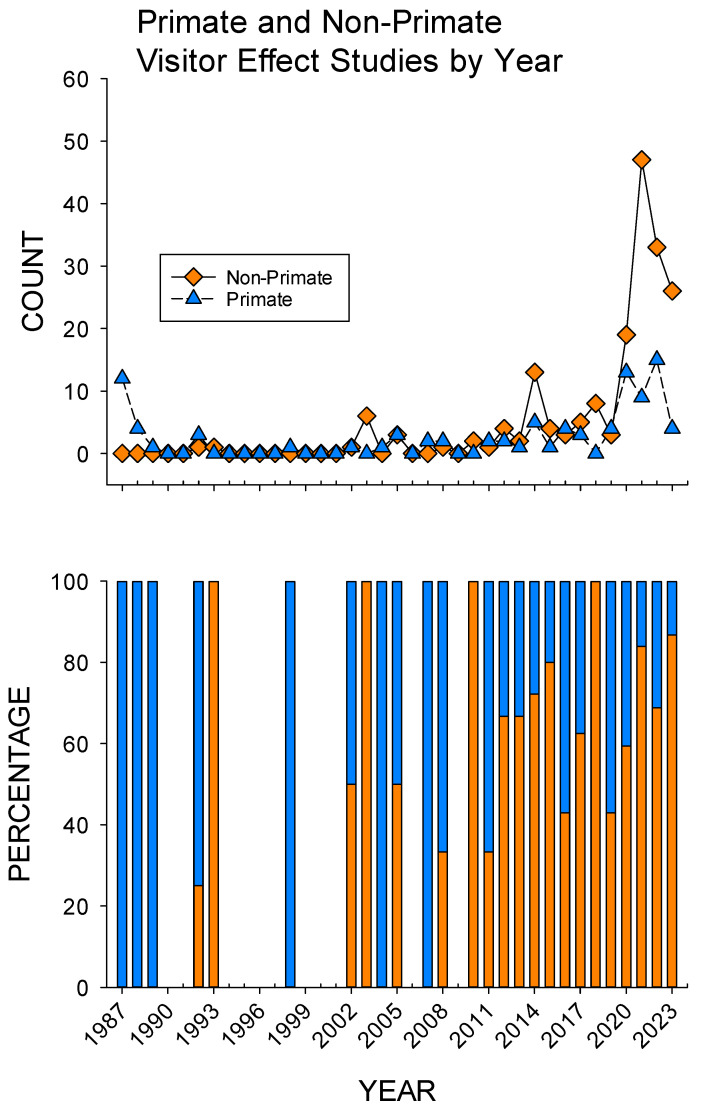
Overview of the changes in primate and non-primate visitor effect studies published per year from 1987 to 2023. Note: orange = non-primate studies; purple = primate studies.

Figure 4 shows the count and percentage of primate and non-primate visitor effect studies by year. Until 2010, the majority of visitor effect studies focused on primates. The 2010 and 2020 increases in visitor effect studies also came with an increase in non-primate species studied. In testing our second hypothesis that non-primate visitor effect studies increased over time proportionally compared to primate visitor effect studies, we compared the earlier period (1987–2010, *n* = 13) to the later period (2011–2023, *n* = 13), with a significant difference demonstrated (*U* = 44.0, *p* = 0.039). Overall, non-primate visitor effect studies increased proportionally from a mean of 35.3% (SE = 11.5) in the earlier period to a mean of 66.6% (SE = 5.3) in the later period.

### 3.5. Positive and Negative Welfare Impacts in Visitor Effect Studies

Out of the 277 visitor effect studies, 119 studies had interpreted a combination of negative, positive, or neutral impacts on animal welfare, and a total of 416 separate visitor effect outcomes were recorded. Neutral outcomes were the majority (59.4%, *n* = 247), around one-third of the outcomes were negative (29.8%, *n* = 124), and one-tenth were positive (10.8%, *n* = 45). Overall, significantly more negative than positive impacts on animal welfare were recorded (χ^2^ = 34.673, *df* = 1, *p* < 0.001).

**Figure 5:**
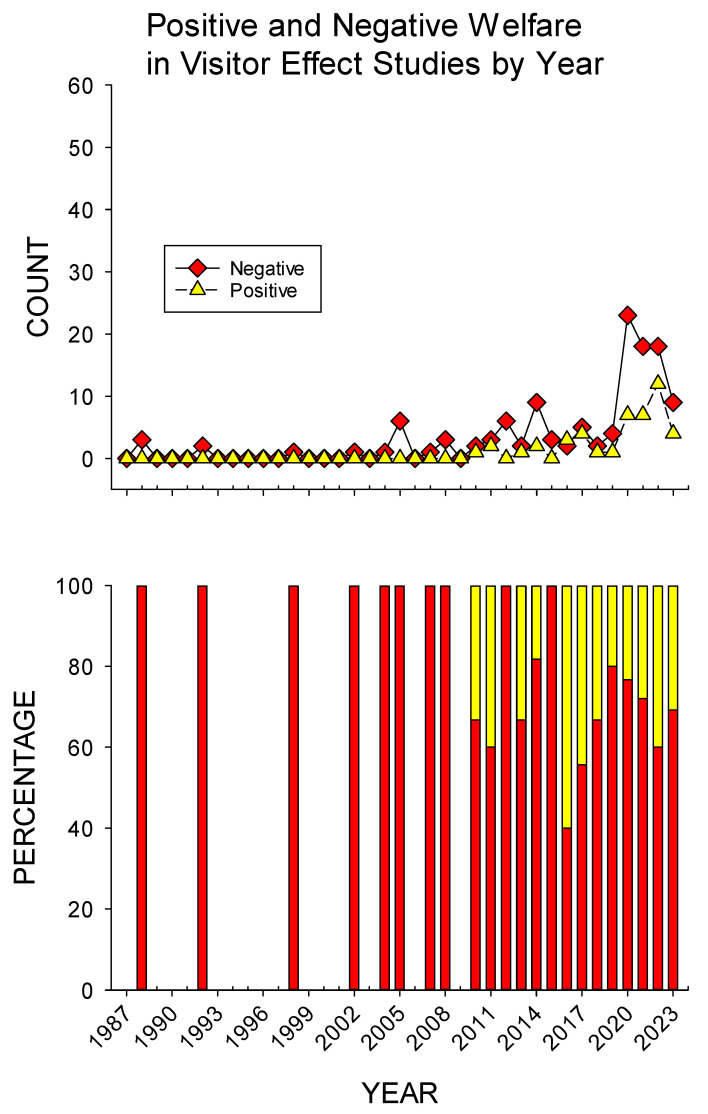
Overview of the changes in positive and negative welfare impact measures in visitor effect studies published per year from 1987 to 2023. Note: red = negative welfare impact; yellow = positive welfare impact.

Figure 5 shows the count and percentage of positive and negative welfare impacts in visitor effect studies by year (as noted earlier, neutral effects were observed in ~90% of all visitor effect studies and therefore not included in further analyses). Until 2010, visitor effect studies only measured negative welfare impacts. The 2010 increase in visitor effect studies also came with an increase in the positive welfare impacts measured. From 2010 to 2023, there was a decreasing trend in the frequency of negative welfare impacts measured and an increasing trend in the frequency of positive welfare impacts measured. In testing our third hypothesis that positive welfare impacts measured in visitor effect studies increased over time proportionally compared to negative welfare impacts measured in visitor effect studies, we compared the earlier period (1987–2012, *n* = 11) to the later period (2013–2023, *n* = 11), with a significant difference demonstrated (*U* = 20.0, *p* = 0.006). Overall, positive welfare impacts measured in visitor effect studies increased proportionally from a mean of 6.7% (SE = 4.5) in the earlier period to a mean of 30.1% (SE = 4.7) in the later period.

#### Taxa and Positive or Negative Welfare Impacts in Visitor Effect Studies

Table 3 shows the different classes of animals and the measures of negative or positive welfare impacts in visitor effect studies observed. A significant portion of visitor effect studies focused on mammals (77.6%, *n* = 214) (χ^2^ = 580.552, *df* = 4, *p* < 0.001). Birds were the next most-studied animals (13.4%, *n* = 37), followed by reptiles (3.6%, *n* = 10) and amphibians (2.2%, *n* = 6). Fish had the fewest visitor effect studies to date (1.8%, *n* = 5).

Overall, all animal classes studied had more instances of negative than positive welfare impacts measured. Fish had the lowest frequency of negative welfare impacts measured (66.7%). Amphibian visitor effect studies only recorded negative welfare impacts.

### 3.6. The Visitor Experience and Impact on Visitors

As previously noted, within AVIs, the visitor experience describes the impact of animals on visitors. We also coded the impact recorded for visitor experience studies (positive, negative, and neutral), which, like visitor effects, were based on author interpretations. Of the 37 visitor experience studies, none indicated a negative impact on visitors. Most studies (45.9%, *n* = 17) reported a combination of positive and neutral visitor impacts. Positive-only visitor impacts were observed in 16 visitor experience studies (43.2%), while neutral-only visitor impacts were observed in 4 visitor experience studies (10.8%).

## 4. Discussion

The objective of this systematic review was to identify changes in the key factors of AVIs over time. More specifically, this paper provides a systematic review of changes that have occurred in AVI publications with respect to visitor effect and visitor experience studies, primate and non-primate visitor effect studies, and positive and negative welfare impacts observed in visitor effect studies. Overall, we found that although the proportion of visitor effect and experience studies has stayed relatively the same, both have continued to grow over time. In addition, the focus of most visitor effect studies has largely appeared to shift away from primates to non-primate species, as well as toward greater demonstrations of positive welfare impacts on the exhibited animals. We consider the implications of these changes below, as well as other AVI-relevant factors.

### 4.1. Publications over Time

AVI research has steadily increased throughout the years, seeing an increase in the regularity of publications in 2010 and a massive total publication increase in 2020. The average number of papers increased by eight-fold annually starting in 2020 compared to the average from 1987 to 2019.

The surge in publications between 2020 and 2023 coincided with the global COVID-19 pandemic, potentially due to opportunities for AVI research during this period [11]. The pandemic led to nationwide zoo lockdowns, offering a unique opportunity to compare visitor effects on animals pre-, during, and post-COVID [30,31,32,33], as well as studying multiple species concurrently [23,24,34,35,36]. However, only 20 of the 82 papers published between 2020 and 2023 involved data collection during COVID-19 (see Table 2). This suggests that the surge in AVI publications that started in 2020 may have been the result of researchers having more time to work on manuscripts due to lockdowns that kept them at home and out of temporarily closed zoos. In addition, the increase could have been influenced by the growing trend in increased peer-reviewed publications for all fields, including zoo welfare and AVI research. Regardless, although the pandemic may have facilitated an increased number of AVI publications, the more recent exponential growth in AVI publications suggests alternative factors being involved, including indirect pandemic-related factors (i.e., more time to publish manuscripts) or non-pandemic-related factors altogether, such as an increased interest in AVI research.

Regardless of the reasons for this exponential growth in AVI research, the reality is that this increase has occurred and will likely continue to occur. As such, it is imperative that future AVI researchers consider what is most important to study, both as it relates to what we learn and how to manage zoo animals and their visitors. Considerations of factors such as the time invested in studying the impact of interactions on visitors as well as animals, the exhibited species of focus, and the welfare measures utilized to study the impact of any interactions are all worth considering as we plan for future research.

### 4.2. Visitor Interactions over the Years

AVIs have traditionally focused on visitor effects to understand the influence humans have on animals [5,6]. Although our first hypothesis regarding the proportional increase in visitor experience studies compared to visitor effect studies over time was not supported (a near significant effect of *p* = 0.053; a proportional increase from 11 to 13% of the overall AVI research), there does appear to be an increase in more AVI visitor experience research (see Figure 3). This is emphasized by the fact that 19 of the 23 visitor experience papers (26 of 37 visitor experience studies) included in this review were published in the last decade.

With the steady increase in AVI-based visitor experience research, we have also witnessed continued expansion in its topics of interest. In one of the first reviews of the AVI visitor experience research, Godinez and Fernandez [12] noted the lack of literature surrounding the field of visitor experiences when comparing variables such as conservation efforts and zoo perceptions to non-visitors. The first AVI-focused visitor experience papers were published in 2003, focusing on visitor perceptions to enhance zoos’ education, conservation, and research efforts [25,37]. Subsequent visitor experience papers from 2013 on continued to examine multiple visitor experience-related factors, including education and conservation [20,27,29,38,39]. A 2016 publication marked the first study measuring changes in human health parameters in relation to zoo animals [40]. This demonstrates how visitor experience publications can potentially expand beyond the area of AVIs and into other fields of research. Most recently, the first book on human–animal interactions (HAIs) in zoos and aquariums was published [16], giving equal time to both visitor effect and visitor experience chapters. Additionally, last year a meta-analysis of the effects of visiting zoos and aquariums was published, suggesting that zoo visits, which include animal–visitor interactions, were correlated with increases in conservation beliefs and behaviors [14]. Therefore, zoo-based visitor experience research has spread across multiple areas of interest and fields, demonstrating that AVI research can play a critical role in improving our understanding and ability to improve visitor education and conservation action in and out of the zoo.

What is additionally needed is research aimed at ways to modify visitor behavior in a manner that better promotes the welfare of exhibited animals while simultaneously promoting positive visitor experiences. For instance, Blaney and Wells [41] previously noted that by providing camouflaged netting to make visitors less visible to gorillas, it also altered visitor behavior in a way that possibly benefited the visitor experience, with parents imploring children to be quieter in the ‘jungle’. More recently, Tay et al. [42] found that visitors were more likely to alter and avoid potentially negative interactions with animals in the presence of staff but not in the presence of signs. It is critical that our future AVI research not only includes more visitor experiences but is also more inclined to understand the best ways to manage visitor interactions.

### 4.3. Diversification of Taxa in Visitor Effect Studies

AVI research in the area of visitor effects shows an uneven distribution across animal classes, with a focus on mammals and birds, while studies on reptiles, amphibians, and fish have been limited, particularly before 2020. Similar taxonomic variations in AVIs offered by zoos and aquariums were noted by D’Cruze et al. [21]. This preference may contribute to the concentration of visitor effect studies on mammals. Furthermore, previous reviews alluded to a disproportionate number of visitor effect studies on primate species [4,5,6,9]. Although this primate focus was true of earlier AVI research, our findings support our second hypothesis that, proportionally, the number of non-primate visitor effect studies has increased (see Figure 4), which coincides with the review findings of Williams et al. [11]. Nonetheless, there still appears to be a limited number of non-mammalian AVI research published, particularly with respect to positive welfare measures (see Table 3). Future visitor effect studies should look to examine a wider array of taxa in zoos, since different species, particularly ones from distinctly non-mammalian species, are likely to have different responses to the presence of visitors. This is also true of individuals within a species, since different animals in different settings at different times can respond differently to the same conditions. Therefore, visitor effect studies should necessarily look to better understand how a variety of taxa, particularly non-mammalian species, as well as different individuals, are likely to respond to visitor interactions.

### 4.4. Impact of Visitor Effects on Animal Welfare

Historically, negative impacts on animal welfare (i.e., negative visitor effects) were more frequently measured than positive visitor effects (see Figure 5). More recently, this has changed, supporting our third hypothesis that measures of positive welfare in visitor effect studies have increased proportionally and over time compared to negative welfare measures in visitor effect studies. This trend may be attributed to the improved management of captive zoo animals or the increasing use of positive welfare indicators in research. Early measures for assessing animal welfare primarily focused on identifying negative indicators of welfare, such as aggression or repetitive, stereotyped behaviors [7,9,43]. The measurement of positive welfare indicators is a critical component of applied animal research [44,45]. This trend may also suggest a growing interest in increasing positive animal–visitor interactions in zoos, given that both direct and indirect interactions are regularly scheduled events for most zoos around the world [21], and more recent AVI reviews have focused on positive interactions [9,11]. Finally, the increased use of positive welfare measures in visitor effect studies appears to coincide with a shift toward non-primate visitor effect research (see Figure 4). As such, the previous focus on measuring negative welfare impacts may have been concomitant with research focused on primates, particularly apes, which as a taxon may respond more poorly to visitor activity and presence [18,46,47,48,49]. Regardless, from a management perspective, future visitor effect studies focused on positive welfare indicators are critical, as they help better identify ways to manage both animals and visitors in a manner that best promotes such interactions.

### 4.5. Limitations

The positive and negative interactions measured in this review relied on author interpretations. Although those interpretations required quantified and directional, statistically significant change to be included in our review, they still relied on the value judgements placed on the occurrence of some behavioral event by the researchers recording those events. As such, recent changes in increased positive welfare impacts observed in visitor effect studies (as well as the historically high negative welfare impacts observed) could partially be due to experimenter bias for such observations. In addition, any results could be based on the welfare impacts observed for one individual or as an average result across a number of individuals or sessions. Simply stated, a positive or negative welfare impact need not necessarily mean that any individual at all times or all individuals at any one time responded to visitors as such.

The majority of AVI research has been correlational, making it challenging to determine whether the observed effects are due to the animal on the visitor or vice versa. Although correlational studies are common in studying visitor effects in zoos [9], they have limitations, including the inability to control potential confounding variables and difficulty in interpreting results. More recent AVI publications have shown that relying solely on visitor variables can lead to assumptions of a visitor effect when external variables such as time of day, season, and weather have a greater impact on changes in animal behavior [50,51]. As suggested by Goodenough et al. [50], measuring environmental variables alongside visitor variables should be considered in future research to prevent overestimating the impact visitors have on the behavioral variability of captive animals.

### 4.6. Future Directions for AVI Research

Behavioral indicators are commonly used to assess animal welfare in visitor effect studies, often using ethograms. In the process of reviewing publications, it was noted that non-species-specific ethograms were occasionally used, especially in multi-species research. For example, Boyle et al. [23] used a single general ethogram for mammals to observe multiple species in different taxonomic orders (carnivores and primates). Similarly, Frost et al. [35] used a single pre-determined ethogram to observe multiple species of mammals and birds. It is known that species and age can affect an animal’s response to a reduction in its welfare [45]; therefore, the use of species-specific ethograms is encouraged. Despite advancements in the development of species-specific and standardized welfare assessments [52,53,54,55,56], there is a need to identify and validate additional indicators that comprehensively cover animal welfare for more zoo species, in particular for less-studied taxa. With the growing recognition of the significance of positive welfare in assessments, the indicators of positive welfare should be included as well.

The welfare indicators of visitor effects are also typically limited to the ethogram metrics noted above. However, multiple measures of welfare, including the use of enclosure-use indices [57,58] and behavioral diversity [59,60], could help better understand the impact of visitors on animals. Likewise, the use of metrics beyond simply visitor presence or absence, including the type of visitor activity and volume, could facilitate our understanding of visitor effects. Similarly, metrics beyond visitor activity or survey responses should facilitate our understanding of the visitor experience. For example, Fukano et al. [61] used internet activity, including Twitter posts, to better understand how the general public responded to the debut of Japanese rock ptarmigan (*Lagopus muta japonica*) in Japanese zoos. The critical point is that an increased use of metrics ensures not only that actual visitor effects and experiences are better captured but also that more zoos can engage in using at least some of these metrics when trying to better evaluate the impact of AVIs within their facilities.

Correlational studies, although common in AVI research, do not explain causality. Experimental research manipulating visitor variables in a controlled manner would be required to determine causality [9,15]. Examples of publications using this approach have controlled for variables such as visitor viewing access [62,63], visitor visibility via visual barriers [41,64], and visitor noise levels [46]. In addition, the impact of overlooked variables and their influence on interactions is also an area that should be considered in future experiments. For example, the impact of a new seal introduction on both visitors and exhibited seals [65].

There is also a need for future research and practice to focus on promoting positive interactions for both animals and visitors simultaneously. AVIs have demonstrated positive benefits for both zoo animals and visitors during activities such as public feedings and petting zoos [15,66,67,68,69]. The existence of AVIs in zoos, both direct and indirect, has been well-documented as important events when appropriately planned [21]. AVIs can and should be utilized to simultaneously promote positive animal welfare and enhance visitor education in zoos and aquariums. This is particularly true for events such as animal performances or shows, where interactions with animals are specifically scripted to improve visitor education and entertainment while ideally providing a rewarding experience for the animals [70,71].

## 5. Conclusions

This review highlights a growing interest in AVI research, particularly in the past several years. In that time, AVI research has increasingly changed focus from primates to non-primate species, as well as shown greater interest in measuring positive impacts on animals and visitors alike. Future enhancements to AVI research should involve more experimental manipulations, greater consideration of non-interaction-specific factors, such as new animal introductions, and emphasize promoting animal- and visitor-positive interactions. In addition, the establishment of more standardized species-specific welfare assessments should help improve AVI research. We hope that this review will serve as a guide for future science and application, helping to balance visitor effect research that focuses on the welfare of captive animals with visitor experience research that focuses on the educational, conservation, and entertainment objectives of zoos and aquariums.

## Figures and Tables

**Table 1:** Papers unrelated and related to COVID-19.

	Unrelated to COVID-19		COVID-19-Related	
	Count (*n*)	Proportion (%)	Count (*n*)	Proportion (%)
**2020**	20	100.0	0	0.0
**2021**	14	63.6	8	36.4
**2022**	10	50.0	10	50.0
**2023**	18	90.0	2	10.0

**Table 2:** Summary of visitor effect and experience papers and studies.

	Papers		Studies	
	Count (*n*)	Proportion (%)	Count (*n*)	Proportion (%)
**Visitor** **Effects**	139	88.5	277	88.2
**Visitor** **Experiences**	23	14.6	37	11.8
**Total**	157		314	

Note: a total of 157 papers were included, with 5 papers looking at both visitor effects and experiences, therefore resulting in a paper sum > 157 (*n* = 162).

**Table 3:** Overview of negative and positive welfare outcomes across taxonomic classes.

	Negative		Positive	
	Count (*n*)	Proportion (%)	Count (*n*)	Proportion (%)
**Mammal**	96	72.2	37	27.8
**Bird**	14	73.7	5	26.3
**Amphibian**	6	100.0	0	0.0
**Reptile**	6	75.0	2	25.0
**Fish**	2	66.7	1	33.3

## Data Availability

Overview of reviewed papers and data available upon request.
